# *Bifidobacterium longum* Subspecies *infantis* (*B. infantis*) in Pediatric Nutrition: Current State of Knowledge

**DOI:** 10.3390/nu12061581

**Published:** 2020-05-28

**Authors:** Maciej Chichlowski, Neil Shah, Jennifer L. Wampler, Steven S. Wu, Jon A. Vanderhoof

**Affiliations:** 1 Nutrition Science, Department of Medical Affairs, Mead Johnson Nutrition, Evansville, IN 47721, USA; Neil.Shah@rb.com; 2 Clinical Research, Department of Medical Affairs, Mead Johnson Nutrition, Evansville, IN 47721, USA; Jennifer.Wampler@rb.com (J.L.W.); Steven.Wu2@rb.com (S.S.W.); 3 University College London, Great Ormond Street, London WC1N 3JH, UK; 4 Division of Pediatric Gastroenterology, Indiana University School of Medicine, Indianapolis, IN 46202, USA; 5 Boston Children’s Hospital, Gastroenterology, Boston, MA 02115, USA; Jon.Vanderhoof@childrens.harvard.edu

**Keywords:** *Bifidobacterium longum* subspecies *infantis*, *B. infantis*, probiotics, pediatric nutrition, human milk oligosaccharides, inflammation, gut health, microbiome, short chain fatty acids, acetate

## Abstract

Since originally isolated in 1899, the genus *Bifidobacterium* has been demonstrated to predominate in the gut microbiota of breastfed infants and to benefit the host by accelerating maturation of the immune response, balancing the immune system to suppress inflammation, improving intestinal barrier function, and increasing acetate production. In particular, *Bifidobacterium longum* subspecies *infantis* (*B. infantis*) is well adapted to the infant gut and has co-evolved with the mother-infant dyad and gut microbiome, in part due to its ability to consume complex carbohydrates found in human milk. *B. infantis* and its human host have a symbiotic relationship that protects the preterm or term neonate and nourishes a healthy gut microbiota prior to weaning. To provide benefits associated with *B. infantis* to all infants, a number of commercialized strains have been developed over the past decades. As new ingredients become available, safety and suitability must be assessed in preclinical and clinical studies. Consideration of the full clinical evidence for *B. infantis* use in pediatric nutrition is critical to better understand its potential impacts on infant health and development. Herein we summarize the recent clinical studies utilizing select strains of commercialized *B. infantis*.

## 1. Introduction

The universal importance of bifidobacteria in human microbiota and gastrointestinal (GI) health from infancy to advanced years (as reviewed [1]), including the critical role of genus *Bifidobacterium* in the process of immune maturation early in life (as reviewed [2]), have been detailed in a wealth of literature. Bifidobacteria are Gram-positive, heterofermentative, anaerobic bacteria with a distinctive bifid (i.e., “Y”) shape [3]. Genus *Bifidobacterium* was originally isolated from the stool of breastfed infants in 1899, by Henri Tissier (as reviewed [2]). Bifidobacteria colonize the newborn gut within the first days and weeks after birth and represent the most abundant bacterial genus ranging from 40% to 80% of the total gut microbiota [4,5]. Vertical transmission of bifidobacteria from the mother (vagina, GI tract, or breast milk) has been demonstrated [6]. Vaginal delivery provides a higher abundance of *Bifidobacterium* spp. in infants compared to caesarean section (C-section) delivery [7,8]; however, the differences in the gut microbiomes of C-section and vaginally delivered neonates are not apparent until day five of life [9]. Further, differences in *Bifidobacterium* spp. colonization between vaginal and C-section delivery diminish by 30 days of age [10], highlighting the first month of life as a critical period to establish colonization. Reduced abundance of *Bifidobacterium* spp. in infants has been correlated to chronic diseases, including asthma and obesity [11], as well as to lower vaccine response [12]. Henrick and colleagues postulated that loss of *Bifidobacterium* spp. in the infant gut in populations of developed countries is linked to increased incidence of allergic and autoimmune diseases [13]. *Bifidobacterium* spp. abundance can be further influenced by nutrition, antibiotic use, and puberty [14,15,16]. *Bifidobacterium longum* subspecies *infantis (B. infantis)* in particular, dominates the gut microbiota of breastfed infants and benefits the host by accelerating maturation of the immune response, balancing the immune system to suppress inflammation, improving intestinal barrier function, and increasing acetate production [17]. This symbiotic relationship is an example of coevolution (humans and *B. infantis*) to protect the full term neonate and nourish a healthy gut microbiota prior to weaning (as reviewed [18]).

Here, we focus on the current status of knowledge for *B. infantis*, including the preclinical data and clinical evidence.

## 2. Current Status of Knowledge

### 2.1. Carbohydrate Metabolism and Short Chain Fatty Acids (SCFA) Production

**Figure 1:**
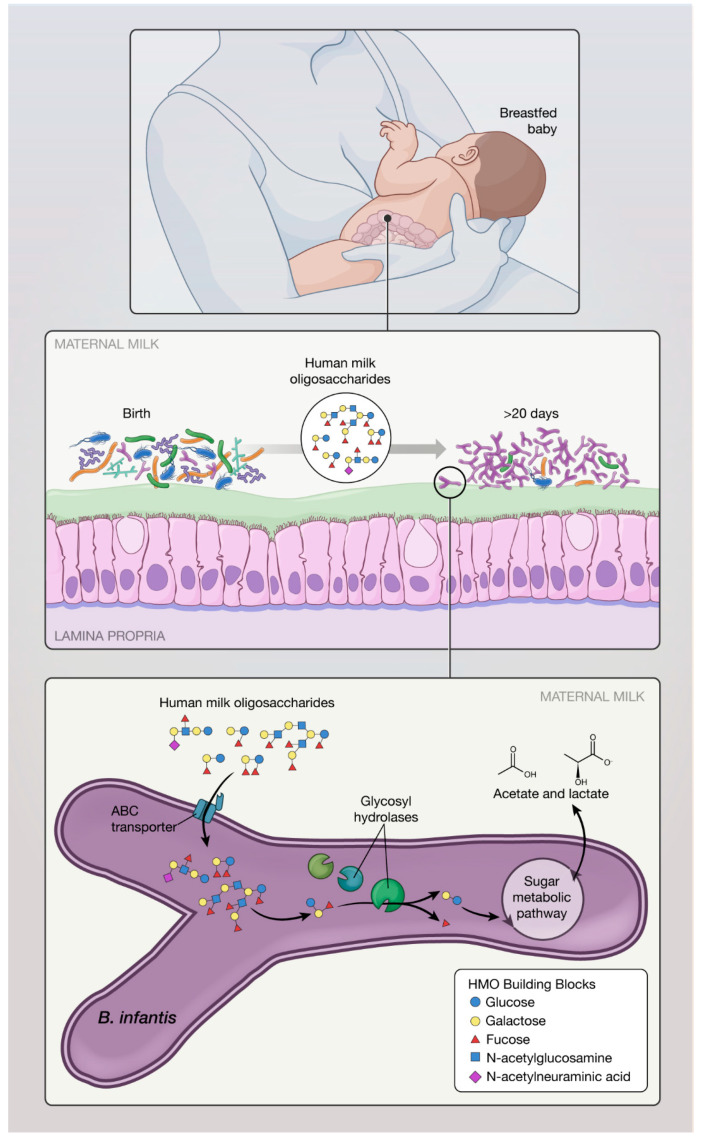
During breastfeeding, infant ingests human milk oligosaccharides (HMO) which allow selective growth of *B. infantis* in the lower gut. *B. infantis* thrives and dominates the gut of breastfed infant, providing numerous benefits important in healthy development. *B. infantis* is evolutionary unique in its ability to metabolize HMO and produce short chain fatty acids (SCFA).

**Figure 2:**
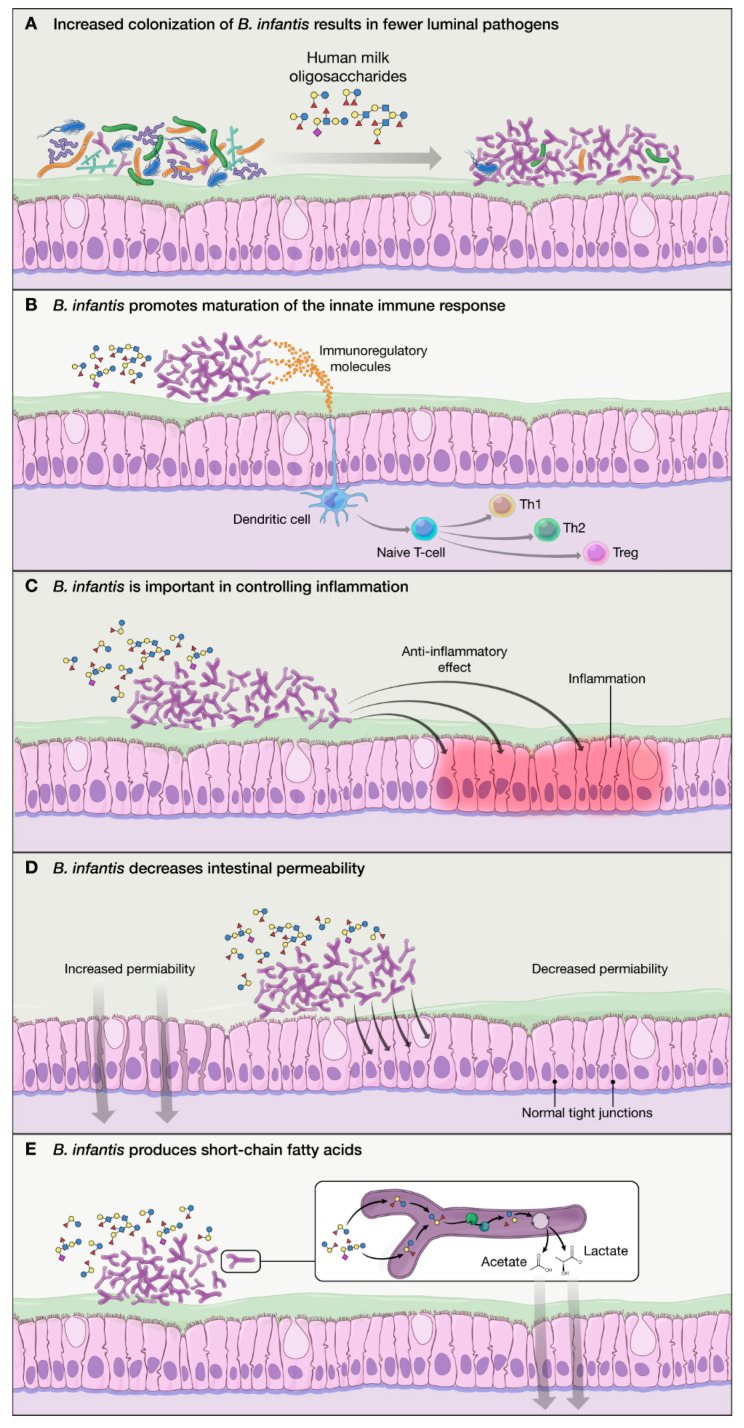
Mechanisms of action of *B. infantis*: (**A**) *B. infantis* is equipped in “enzymatic machinery” to metabolize HMO, allowing increased colonization and fewer luminal pathogens via competitive advantage; (**B**) *B. infantis* produces exogenous substances that promote maturation of the immature innate immune response in breastfed infants; (**C**) *B. infantis* has anti-inflammatory properties benefiting the epithelial layer in the gut; (**D**) *B. infantis* reduces intestinal permeability and helps with “leaky gut”; and (**E**) *B. infantis* produces SCFA (particularly acetate) which have a beneficial effect on the host.

Human milk oligosaccharides (HMO) are the primary substrate for *B. infantis* metabolism (as reviewed [19]). HMO in human milk cannot be metabolized by the infant or by the majority of bacteria in the infant gut, which lack the required enzymes necessary to access and metabolize complex HMO. *B. infantis* demonstrates growth in vitro using HMO as the sole carbon source, reaching a cell density 3-fold higher than *B. longum* subsp. *longum*, *B. breve*, *B. bifidum*, and *B. adolescentis* [20,21]. Once consumed by the infant, intact HMO transit through the infant stomach and proximal small bowel, and are transported directly into the cytoplasm of *B. infantis* (Figure 1) to activate many genes involved in catabolism of HMO (“HMO cluster”) [22]. Compared to other *Bifidobacterium* species, the ability of *B. infantis* to sequester and facilitate complete digestion of HMO (via expression of up to 16 glycosyl hydrolases, such as α-fucosidases, β-galactosidases, β-hexosaminadases, and α-sialidases) [22,23,24] provides a competitive advantage over other GI microbiota, including pathogens (Figure 2A). Genes encoding carbohydrate transporters present in *B. infantis* also contribute to protection against *Escherichia coli* O157:H7 [25]. Of importance, HMO utilization genes are conserved across all *B. infantis* strains [26]. *B. infantis* preferentially consumes smaller HMO species (degree of polymerization <7) which are consistently produced over the course of lactation and represent the bulk of HMO present in pooled milk samples [27]. HMO metabolism by *B. infantis* produces short chain fatty acids (SCFA), such as acetate, which provides an important role in nutrition and intestinal and immune development, facilitates direct binding to intestinal cells, and stimulates anti-inflammatory/inhibits pro-inflammatory cytokine release by intestinal cells [3,28,29]. Acetate produced by bacteria acts in vivo to promote the defense functions of host epithelial cells [25]. Further acetate produced by *B. infantis* becomes a carbon source that stimulates growth and function of butyrate-producing microbes [30]. Thus, HMO stimulate the growth of bifidobacteria and by cross-feeding increase the production of butyrate, a preferred energy source for colonocytes [31]. In addition to their role in the gut, SCFA produced by *B. infantis* can enter circulation and directly affect the adipose tissue, lungs, brain, and liver, inducing overall beneficial metabolic effects [31]. For example, a small fraction of acetate crosses the blood–brain barrier, where it is taken up and activates hypothalamic neurons driving satiety [32], suggesting that acetate has a direct role in central appetite regulation. Lactate also was shown to cross blood–brain barrier and functions as an energy substrate of the brain [33]. 

Whereas other bacteria consume HMO (e.g., Bacteroidaceae), only Bifidobacteriaceae converts HMO to acidic end products that affect stool pH [13]. Therefore, acidic fermentation of selected HMO by *B. infantis* may result in fecal pH changes and reduction of dysbiotic taxa in the gut. Preclinical models, including the necrotizing enterocolitis (NEC) model, have demonstrated that *B. infantis* could potentially decrease intestinal permeability and increase stabilization of tight junction proteins [28,34]. For example, indole-3-lactic acid (ILA) is an anti-inflammatory molecule which significantly attenuates lipopolysaccharide-induced activation of NF-κB in macrophages; it also attenuates an increase in the pro-inflammatory cytokine IL-8. *B. infantis* contributes to maintaining the intestinal barrier integrity through ILA, a metabolite of tryptophan [35], and may protect the epithelium via activation of the aryl hydrogen receptor, which can further promote normal intestinal immune function. *B. infantis* could also potentially protect against excessive intestinal inflammation which often affects the premature infant and may be a contributing factor in NEC.

### 2.2. Proposed Mechanisms of Action of B. infantis Based on In Vitro Studies:

- *B. infantis* is highly specialized for the consumption of HMO and has a competitive advantage against other bacteria, allowing increased colonization and resulting in fewer luminal pathogens (Figure 2A) [36].
- *B. infantis* produces exogenous substances that promote maturation of the immature innate immune response (Figure 2B) [28].
- HMO “turn on” the repertoire of genes in *B. infantis* that are important in controlling inflammation within the infant gut (Figure 2C) [22].
- *B. infantis* becomes dominant in the gut and reduces pH by its unique ability to metabolize all HMO into acidic end products, lactate, and acetate (Figure 2E) [36].
- *B. infantis* improves the intestinal barrier integrity through the production of tryptophan metabolite, indole-3-lactic acid [35].

### 2.3. Clinical Evidence: Safety and Efficacy of Select Commercialized Strains of B. infantis Used in Pediatric Populations

In 2007, the European Food Safety Authority (EFSA) assigned qualified presumption of safety (QPS) status to the bacterial species *B. longum*, which includes subspecies *infantis*, indicating that this taxonomic group does not raise any safety concerns [37]. The QPS status, which applies to all strains of *B. infantis* listed in this review, indicates that none of those strains have been associated with human clinical disease. In addition, based on our knowledge, *B. infantis* has not been associated with any reports on antibiotic resistance. Here, we summarize clinical evidence for safety and efficacy of select commercialized *B. infantis* strains used in pediatric populations (Table 1).

#### 2.3.1. *B. infantis* M63 

Term infants identified with colic were enrolled (*n* = 66) in a multicenter double-blind randomized controlled trial (DBRCT); participants received a standard infant formula (IF) or an IF with added probiotics (10 million CFU *B. infantis* M63 per 100 mL formula (Morinaga, Japan)) and 10 million CFU *Lactobacillus rhamnosus* LCS742 per 100 mL formula [38]) over a one month feeding period. Feeding-related GI adverse events were significantly lower in infants receiving M63. The same probiotic mix was used in another DBRCT trial, where term infants (*n* = 97; <postnatal day (PND) three of age) received M63 and LCS742 (140 million CFU per 100 mL formula, each) for six months [39]. After one month of feeding, infants receiving IF with added probiotics exhibited less crying or agitation and more quiet behavior (*p* = 0.03) and at six months, atopic dermatitis was less frequently observed (*p* < 0.05). Overall, the results from these two studies suggest the potential beneficial effect in a population of children with colic as being improved tolerance and a protective effect against the development of atopic dermatitis.

A probiotic mix of M63 (1 billion CFU/day), *B. breve* M16 (1 billion CFU/day), and *B. longum* BB536 (3 billion CFU/day) has been studied in several clinical trials. For example, children (*n* = 55, 4–12 years of age) with functional constipation received polyethylene glycol (PEG, a laxative) or PEG and the probiotic mix over an eight week period [40]. The probiotic mix was demonstrated as safe; however, no difference in efficacy was demonstrated between groups. In a placebo-controlled study, children (*n* = 40; 9 ± 2.2 years of age) with allergic rhinitis and asthma received the same probiotic mix over a four week period [41] and showed significant improvement of symptoms. In a DBRCT crossover trial, children (8–16 years of age) with irritable bowel syndrome (IBS) (*n* = 48) and functional dyspepsia (*n* = 25) received the same probiotic mix over a six week period [42]. Administration of probiotics improved abdominal pain in children with IBS but not with functional dyspepsia. The proportion of IBS children who reported an improvement in QoL (quality of life) was significantly higher after probiotics than after placebo (48% vs. 17%). In a trial conducted in Japan (*n* = 44, six weeks old), low-birthweight infants assigned to a modified probiotic mix of M63, M16, and BB536 (500 million CFU/day of each strain) over a six week feeding period demonstrated higher bifidobacteria GI colonization compared to those receiving M16 alone [43]. The above studies used a probiotic mix including *B. infantis* M63. Although the benefits cannot be directly attributed exclusively to *B. infantis*, the results are in agreement with several mechanisms of *B. infantis* described above. 

#### 2.3.2. *B. infantis* ATCC 15697

A study by Underwood et al. [44] consisted of two phases; in phase one, premature infants (*n* = 12, five weeks old) received formula feedings and were randomized to receive *B. infantis* ATCC 15697 (4 billion CFU twice daily) or *B. animalis* subsp. *lactis* with doses increased over a five week feeding period. In phase two, nine premature infants receiving their mother’s milk received each of those two bifidobacteria for two weeks separated by a one-week washout period. Fecal bifidobacteria count was significantly higher and, in case of phase two, *Proteobacteria* were significantly lower in the ATCC 15697 group, with authors concluding that ATCC 15697 was more effective at colonizing the premature infants compared to *B. animalis* sbsp. *lactis* [44]. The study also demonstrated that the combination of human milk and *B. infantis* was most effective at “normalizing” the fecal microbiota confirming the specialized ability of *B. infantis* to metabolize HMO. 

Infants with confirmed gastroschisis were enrolled (*n* = 24, gestational age at birth > 34 weeks) in a placebo-controlled pilot study. Participants were randomized to receive either ATCC 15697 (1 billion CFU) or a placebo twice daily for six weeks or until hospital discharge [45]. ATCC 15697 was well tolerated, even during the period of gastric suctioning. Infants fed *B. infantis* had higher fecal Bifidobacteriaceae, lower Clostridiaceae, and trends toward lower Enterobacteriaceae, Enterococcaceae, Staphylococcaceae, and Streptococcaceae. Clinical outcomes, including length of hospital stay did not differ between groups.

#### 2.3.3. *B. infantis* UCD272

Children with autism spectrum disorders (ASD; *n* = 11, 2 to 11 years of age) and a history of chronic constipation, diarrhea, or IBS were enrolled in a crossover study; participants received a combination of *B. infantis* UCD272 (20 billion CFU/day; Culture Systems, Inc., Mishawaka, IN) and a prebiotic (bovine colostrum product) over a five-week period, followed by a two-week washout, and five weeks of prebiotic alone [46]. UCD272 was well tolerated; however, a trend toward greater reduction in GI symptoms, aberrant behavior and immune imbalance was observed during use of the prebiotic alone. Conclusions were limited due to the small number of participants enrolled.

#### 2.3.4. *B. infantis* EVC001

Clinical outcomes related to *B. infantis* EVC001 in breastfed infants were described in several recent publications. Mother/infant dyads (*n* = 80) were randomized by parallel assignment to either lactation support (LS; Control) or LS + EVC001 (Evolve Biosystems, Davis, CA) over a 21-day feeding period [47]. EVC001 was packaged in single-use sachets (156 mg; 18 billion CFU) and delivered diluted with lactose starting at postnatal day (PND) seven [47]. Primary outcomes included health and safety reporting (body temperature, GI symptom ratings, number of illnesses and sick doctor visits, use of antibiotics or gas-relieving medications, presence of colic, jaundice, flatulence, or bloody stool) and stool *Bifidobacterium* spp. counts from PND 6 to 28. Stool samples were collected through PND 60. In each group, 34 participants were included in the analysis. Stool *Bifidobacterium* spp. count was significantly higher and stool frequency significantly lower in infants receiving EV0001 from PND 6 to 28. No group differences in health and safety outcomes were detected. One month after discontinuing feeding EVC001, stool count of EVC001 persisted and was significantly higher compared to the control group [36]. The dominance of EVC001 influenced beta diversity (diversity between samples); however, there were no differences in terms of microbial species richness (alpha diversity) as the Shannon diversity index was similar between Control and EVC001 groups. Lack of differences in alpha diversity between Control and EVC001 is consistent with previous reports on breast-fed infants. Recently, lower alpha diversity was reported in infants receiving human milk compared to infant formula [59]. In addition, stool HMO from PND 6 to 29 were significantly lower (suggesting increased bifidobacteria metabolism) in the EVC001 group compared to the control group; acetate and lactate were significantly higher in the EVC001 vs. the control group. Increased production of lactate and acetate alters the intestinal environment to prohibit the growth of pH-sensitive pathogenic populations (e.g., Enterobacteriaceae and Clostridia). Specifically, average fecal pH was 5.15 in infants colonized by EVC001, compared to 5.97 fecal pH and 10-fold higher fecal HMO in infants lacking EVC001 colonization [36]. Stool endotoxins were 4-fold lower in the EVC001 group, consistent with observed lower levels of Gram-negative Proteobacteria and Bacteroidetes. The relative abundance of virulent pathogens such as *E. coli*, *Klebsiella*, *Clostridium* and *Staphylocossus* were decreased by over 93% [48]. Shotgun metagenomics was used to examine the effect of feeding EVC001 upon antibiotic resistance genes (ARG, i.e., the resistome) in infants (Control, *n* = 31; EVC001, *n* = 29) from PND 7 to 21 [48]. The resistome is the collection of all the antibiotic resistance genes, including those associated with pathogenic bacteria, non-pathogenic antibiotic producing bacteria, and all other resistance genes [60]. ARG are associated with resistance to a wide range of drugs including β-lactamase, fluoroquinolone, and tetracycline. In the EVC001 group 87.5% fewer ARG were detected in the microbiome; 38 ARG were significantly reduced, and relative and absolute abundance of *Escherichia* (which predominantly harbored ARG) were also significantly reduced [48]. It is possible that infants with fewer ARG could be less likely to exhibit resistance to a wider spectrum of drug classes, however clinical studies are needed to confirm this theory. Further analyses indicated much less evidence of mucous layer erosion and inflammation in infants in the EVC001 compared to the control group. Specifically, using nano-HPLC chip/time-of-flight mass spectrometry, EVC001 was demonstrated to help maintain barrier function by diminishing colonic glycan degradation [49]. In continuation of analyses conducted on the same cohort, significantly more stool N-glycans (specific complex carbohydrates released from milk glycoproteins and available for select use by *B. infantis*) were measured in stool samples from the EVC001 group. The release of selectively fermentable N-glycans may increase the prebiotic activity and increase the growth of EVC001 in vivo. Finally, in stool samples from the same cohort, significantly lower calprotectin and proinflammatory cytokines were reported in infants receiving EVC001 [50] suggesting lower enteric inflammation.

#### 2.3.5. *B. infantis* CECT 7210 

Term infants (<3 months of age) enrolled in a multicenter DBRCT were randomized to receive a standard IF (Control, *n* = 97) or IF with added *B. infantis* CECT 7210 at 10 million CFU per 100 mL formula (Laboratorio Ordesa SL, Spain) (*n* = 93) for a 12-week feeding period [51]. Parent-reported incidence of diarrhea was the primary outcome. Secondary outcomes included incidence of infection, salivary IgA levels, stool microbiota, infant growth, and tolerance measures. A total of 151 infants completed the study (Control, *n* = 78; CECT 7210, *n* = 73). Overall diarrhea events per infant (median; Control: 0.29 ± 1.07, CECT 7210: 0.05 ± 0.28) were not significantly different between groups. Stool frequency was significantly lower in the control group after four weeks of study feeding. By study end, total stool *Bifidobacterium* spp. was similar between groups; however, *B. infantis* was significantly higher in the CECT7210 group. No differences in growth, formula intake, or other secondary outcomes were observed. Overall, the authors concluded that formula with added CECT was safe, effective, and well tolerated in healthy term infants.

#### 2.3.6. *B. infantis* BB02

Jacobs and colleagues studied a mix of three probiotics, including the strain *B. infantis* BB02 in preterm infants (<32 completed weeks of gestation, 1500 g or less) enrolled in a multicenter DBRCT. Infants were randomized to receive daily administration of a probiotic combination (BB02, *Streptococcus thermophilus* TH4, and *B. lactis* BB12; 1 billion CFU/day; Solgar, NJ, USA; *n* = 548) or a placebo (maltodextrin; *n* = 551) [52]. The primary outcome was at least one episode of definite late-onset sepsis. Incidence of NEC (Bell stage 2 or higher) was significantly reduced in infants receiving the probiotic combination; no group difference in definite late-onset sepsis or all-cause mortality was detected. Stool microbiome analysis of the study cohort demonstrated significantly increased *Bifidobacterium* spp. count and reduced genus *Enterococcus* count in infants who received probiotics [53]. Considering that this probiotic combination included other probiotic strains in addition to *B. infantis*, the observed benefits cannot be attributed definitively to BB02. However, the observed increase in *Bifidobacterium* spp. and reduction in NEC are consistent with the mechanisms we described above, e.g., increase of beneficial microbiota and improvement of gut barrier integrity. 

#### 2.3.7. *B. infantis* R0033

Healthy term infants were enrolled (*n* = 208; 3 to 12 months of age) and received *B. infantis* R0033 (3 billion CFU/d; Lallemand Health Solutions, Montreal, Canada) over an eight week feeding period [54]. Other study groups included *L. helveticus* and *B. bifidum*. R0033 was safe, well tolerated, and had no impact on growth (weight, height, and head circumference). A significant decrease in fecal *Blautia, Collinsella*, *Enterococcus* and *Klebsiella* genera and increase in the IL-10/IL-12 ratio were demonstrated in infants receiving R0033, suggesting anti-pathogenic and anti-inflammatory activity [55]. The anti-inflammatory effect of R0033 can be potentially attributed to production of ILA (see “Carbohydrate Metabolisms and SCFA Production” above), although ILA was not measured in this study.

In another DBRCT, children three to seven years of age (*n* = 135) received a daily synbiotic preparation (R0033, *L. helveticus* R0052, *B. bifidum* R0071 (3 billion CFU total), and fructo-oligosaccharide (FOS)) over a three month feeding period [56]. Otherwise healthy, participants suffered from at least three episodes of ear, nose and throat, respiratory tract, or GI illness during the previous winter. Overall, supplementation with a probiotic mix, which included R0033, significantly decreased the risk of occurrence of common infectious diseases in children. No side effects were detected in either group. As in several other studies listed in our review, the associated benefits cannot be exclusively attributed to R003 alone. However, the decrease in infectious diseases, including GI illnesses is supported by well-described mechanisms of *B. infantis*, which include prohibition of pH-sensitive pathogenic organisms.

#### 2.3.8. *B. infantis* BT1

Term infants were enrolled (*n* = 106, newborn) in a DBRCT; participants received a control IF or IF with an added probiotic mix of *B. infantis* BT1, *B. bifidum* BF3, *B. breve* BR3, and *B. longum* BG7 (total 10 million CFU/g) through 12 months of age to measure fecal microbiota diversity and composition [57]. Although *Bacteroides* and *Blautia* spp. counts were lower after one month of feeding in infants receiving probiotics, no group differences in microbiome or metabolite profile were detected after 12 months of age. No significant differences were observed between infant feeding groups regarding growth, antibiotic uptake, or other health variables.

## 3. Conclusions

Studies reviewed in this manuscript suggest that colonization with *B. infantis* might have potential beneficial effects in infants and children. *B. infantis* is well adapted to the infant gut, in part due to its ability to consume complex carbohydrates found in human milk (HMO). As evidenced in clinical studies, the administration of *B. infantis* leads to successful colonization in the gut, where the highly selective and acidic fermentation of HMO results in increased production of lactate and acetate, and subsequent reduction of gut dysbiosis and lower pH. This potentially includes an important role in development and maturation of the immune system. Modern practices, including C-section and perinatal antibiotics have disrupted the mother-to-infant transfer of *B. infantis* at birth, leading to a loss of this key member of the infant gut microbiota, which has resulted in an increase of stool pH in infants [13]. Continuing to gather evidence of the protective effects of *B. infantis* strains within the infant gut will help us better understand the critical role of the infant gut microbiome in establishing long-term health.

## Figures and Tables

**Table 1:** Clinical trials in pediatric populations using select strains of *B. infantis*.

*B. infantis* Strain (Manufacturer)	Trial ID	Site	Enrollment	Feeding Period	Study Design/ Study Groups	Study Outcomes	Conclusions	Year
M63 (Morinaga)	n/a	France	66 infants	1 month	Term infants identified with colic ^a^ at enrollment (3 w to 3 months) multicenter double-blind randomized controlled trial (DBRCT) (1) Standard infant formula (IF) (Control) and, (2) IF + B. infantis M63.	Efficacy and safety parameters at days 15 and 30 (weight and length growth, crying duration).	Feeding-related GI adverse events (e.g., vomiting, constipation, regurgitation, and flatulence) were significantly lower in infant receiving M63.	2010 [38]
	ClinicalTrials.gov NCT00920166	France	97 infants	6 months	Term infants (<postnatal day [PND] 3 at enrollment. Multicenter DBRCT (1) standard IF (Control), and (2) IF + M63. Stool samples collected at 1 and 6 months.	Primary Weight at 6 months. Secondary GI tolerance, manifestation of atopic dermatitis, fecal secretory IgA (SIgA), fecal microbiota composition.	Probiotic diet was safe, well-tolerated and protective against the development of atopic dermatitis. M63 group exhibited less crying or agitation, and more quiet behavior after 1 month of feeding (*p* < 0.02).	2011 [39]
	n/a	Italy	55 children (4–12 years old [yo]) with functional constipation.	8 weeks	Prospective, placebo-controlled, randomized trial (1) polyethylene glycol (PEG; laxative), and (2) PEG + Probiotics: M63 + *B. breve* M16 + *B. longum* BB536.	Frequency of bowel movements, Stool consistency according to the Bristol stool form scale, presence of fecal incontinence, abdominal pain, painful defecation, and rectal bleeding.	PEG with or without added probiotics was equally effective and safe in the treatment of children with chronic constipation. No difference in efficacy among the groups.	2017 [40]
	ClinicalTrials.gov NCT02807064	Italy	40 children (9 yo) with allergic rhinitis and asthma.	4 weeks	Prospective, placebo-controlled, randomized trial (1) probiotics: M63 + M16 + BB536, and (2) placebo.	Primary Assessment of allergic rhinitis. Secondary Quality of life (QoL), frequency and school performance, attention, and evaluation of asthma exacerbations	Children supplemented with M63 showed a significant improvement of symptom score of allergic rhinitis and asthma.	2017 [41]
	ClinicalTrials.gov NCT02566876	Italy	73 children (8–16 yo) with abdominal pain (AP)-associated functional GI disorders (FGID).	6 weeks	Prospective, placebo-controlled, randomized trial (1) probiotics: M63 + M16 + BB536, and (2) placebo	Primary Improvement of frequency and intensity of AP in children with FGID and irritable bowel syndrome (IBS). Secondary Quality of life (QoL).	In children with IBS, a mixture of probiotics was associated with improvement in abdominal pain and QoL.	2017 [42]
	not known	Japan	44 infants (low-birthweight).	6 weeks	(1) M16 (2) probiotic mixture: M63 + M16 + BB536	Composition of the fecal microbiota.	Administration of three species of bifidobacteria resulted in earlier formation of a bifidobacteria-predominant fecal microbiota.	2013 [43]
ATCC 15697	ClinicalTrials.gov NCT00810160	US	12 premature infants.	5 weeks	ATCC 15697 *Bifidobacterium animalis* subsp. *lactis*	Composition of the fecal microbiota.	ATCC 15697 was more effective at colonizing the fecal microbiota in premature infants; fecal bifidobacteria levels were significantly higher in that group.	2013 [44]
	ClinicalTrials.gov NCT01316510	US	24 infants with gastroschisis.	6 weeks (or hospital discharge)	(1) ATCC 15697 (1 billion CFU twice daily) and (2) Control (powdered elemental formula).	Primary Composition of the fecal microbiota. Secondary Length of hospital stay.	ATCC 15697 was well tolerated and had a moderate effect of microbiome. In infants receiving ATCC 15697 there was less dysbiosis, i.e., alterations in the microbiota associated with disease.	2016 [45]
UCD272 (Culture Systems Inc)	ClinicalTrials.gov NCT02086110 (listed as SC268)	US	11 children (2–11 yo) with ASD	12 weeks	DBRC (1) bovine colostrum (prebiotic) + UCD272 20 billion CFU/day, and (2) bovine colostrum	Primary Composition of fecal microbiota. Secondary Serum immune profile.	Conclusions limited due to the small sample size; probiotic supplement was well tolerated. Reduction in IL-13 and TNF-α (proinflammatory cytokines) in some participants.	2019 [46]
EVC001 (Evolve Biosystems)	ClinicalTrials.gov NCT02457338	US	80 mother/ infant dyads	21 days	Randomized, parallel assignment (1) lactation support (LS), and (2) LS + EVC001 ^b^ stool samples collected through PND 60.	Levels of *B. infantis,* incidence of adverse events, and infant weight	Significantly higher stool *Bifidobacterium* spp. in infants receiving EV0001, no differences in health and safety outcomes, and significantly lower daily stool frequency in EVC001 infants.	2017 [47]
						16S rRNA sequencing and mass spectrometry	In infants who received EVC001: significantly higher Bifidobacteriaceae and B. *infantis*, lower stool HMO (suggesting increased metabolism by bifidobacteria), lower stool pH and higher acetate and lactate, and significantly higher stool B. *infantis* for 30 days following end of feeding period.	2017 [36]
						Metagenomics	87.5% fewer antibiotics resistant genes (ARG) detected in the microbiome, and significant reduction in abundance of *Escherichia*.	2018 [48]
						Mass spectrometry	Significantly less colonic mucin-derived O-glycans in stool samples from babies fed EVC001 compared to control. N-glycans (related to milk glycoproteins) increased in EVC001.	2018 [49]
						Immunoassay, 16S rRNA sequencing, ELISA.	Infants receiving EVC001 had significantly lower level of inflammatory cytokines in stool.	2019 [50]
CECT 7210 (IM-1^®^, Ordesa S.L.)	Clinicaltrials.gov NCT02096302	Spain	151 term infants	12 weeks	Multicenter DBRCT (1) Standard IF (Control), and (2) IF + CECT 7210	Primary Changes in diarrhea incidence. Secondary Changes in infections incidence, sIgA, growth, and composition of the fecal microbiota.	In CECT 7210 group: trend for decreased diarrhea reached significance at week 8, and lower constipation incidence. No differences were found in other digestive symptoms, growth, or formula intake.	2018 [51]
BB-02 (Chr. Hansen)	Australia and New Zealand Clinical Trials Register, ACTRN012607000144415	Australia/New Zealand	1099 preterm infants	Through hospital discharge or term corrected age	Multicenter DBRCT (1) Maltodextrin (placebo), and (2) probiotic combination: BB02, *Streptococcus thermophilus* Th4, and *Bifidobacterium lactis* BB12.	Primary At least 1 episode of definite late-onset sepsis. Secondary NEC (Bell stage 2 or more), mortality, length of hospital stay, and weight at discharge.	Incidence of NEC was significantly reduced in infants receiving the probiotic combination but not definite late-onset sepsis or mortality.	2013 [52]
						Composition of the fecal microbiota.	Higher levels of *Bifidobacterium* spp. found in infants who received the probiotics; *Enterococcus* reduced in infants receiving the probiotic mix during the supplementation period	2018 [53]
R0033 (Lallemand)	Clinicaltrials.gov NCT02215304	Spain	221 infants	8 weeks	Multicenter DBRCT (1) Potato starch (placebo), R0033 (3 billion CFU/d), *Bifidobacterium bifidum* R0071, and (2) *Lactobacillus helveticus* R0052.	Primary Weight, height, and head circumference. Adverse events Use of medication. Secondary Urine concentration of D-lactic acid, changes in sleep and crying patterns, and immune compounds in fecal samples.	Use of R0033 was safe and well tolerated. No impact on growth (weight, height, and head circumference), adverse events, or serious adverse events. Increased ratio of IL-10/IL-12 and significant reduction in *Collinsella*, *Enterococcus*, and *Klebsiella* genera in infants receiving R0033.	2017 [54] 2018 [55]
			135 children (3–7 yo)	3 months	Multicenter DBRCT (1) Synbiotic preparation (R0033, *L. helveticus* R0052, *B. bifidum* R0071, and FOS), and (2) placebo.	Percentage of children free of any episodes of ear, nose and throat, respiratory tract, or gastrointestinal illness	Synbiotic preparation decreased the risk of occurrence of common infectious diseases. No side effects were detected in either group.	2010 [56]
BT1	germanctr.de (DRKS00003660)	Germany	106 infants	12 months	Double-blind, randomized, placebo-controlled study (1) BT1, B. bifidum BF3, *B. breve* BR3, *B. longum* BG7 (total 10 million CFU/g), and (2) Control	Composition of the fecal microbiota (16s rRNA sequencing) and fecal metabolome (HPLC).	Probiotic formula modulated the infant stool microbiome (e.g., *Bacteroides*) and metabolome (e.g., lipids) at very early stages of life, with no detectable long-term consequences.	2017 [57]

^a^ colic defined using Wessel criteria [58]; ^b^ from postnatal day (PND) 7.
